# A Single Bout of Aerobic Exercise Improves Motor Skill Consolidation in Parkinson’s Disease

**DOI:** 10.3389/fnagi.2018.00328

**Published:** 2018-10-22

**Authors:** Simon Steib, Philipp Wanner, Werner Adler, Jürgen Winkler, Jochen Klucken, Klaus Pfeifer

**Affiliations:** ^1^Department of Sport Science and Sport, Friedrich-Alexander University Erlangen-Nürnberg, Erlangen, Germany; ^2^Department of Medical Informatics, Biometry and Epidemiology, Friedrich-Alexander University Erlangen-Nürnberg, Erlangen, Germany; ^3^Molecular Neurology, University Hospital Erlangen, Friedrich-Alexander University Erlangen-Nürnberg, Erlangen, Germany

**Keywords:** cardiovascular exercise, motor learning, online learning, offline learning, memory, neuroplasticity, neurorehabilitation, Parkinsonism

## Abstract

**Background:** Motor learning is impaired in Parkinson’s disease (PD), with patients demonstrating deficits in skill acquisition (online learning) and consolidation (offline learning) compared to healthy adults of similar age. Recent studies in young adults suggest that single bouts of aerobic exercise (AEX), performed in close temporal proximity to practicing a new motor task, may facilitate motor skill learning. Thus, we aimed at investigating the effects of a single bout of aerobic cycling on online and offline learning in PD patients.

**Methods:** 17 PD patients (Hoehn and Yahr 1 – 2.5, age: 64.4 ± 6.2) participated in this crossover study. Immediately prior to practicing a novel balance task, patients either performed 30 min of (i) moderate intensity (60–70% VO_2max_) aerobic cycling, or (ii) seated rest (order counterbalanced). The task required patients to stabilize a balance platform (stabilometer) in a horizontal position for 30 s. For each experimental condition, patients performed 15 acquisition trials, followed by a retention test 24 h later. We calculated time in balance (platform within ± 5° from horizontal) for each trial, and analyzed within- and between-subjects differences in skill acquisition (online learning) and skill retention (offline learning) using mixed repeated-measures ANOVA.

**Results:** We found that the exercise bout had no effect on performance level or online gains during acquisition, despite affecting the time course of skill improvements (larger initial and reduced late skill gains). Aerobic cycling significantly improved offline learning, as reflected by larger 24-h skill retention compared to the rest condition.

**Conclusion:** Our results suggest that a single bout of moderate-intensity AEX is effective in improving motor skill consolidation in PD patients. Thus, acute exercise may represent an effective strategy to enhance motor memory formation in this population. More work is necessary to understand the underlying mechanisms, the optimal scheduling of exercise, and the applicability to other motor tasks. Further, the potential for patients in later disease stages need to be investigated. The study was a priori registered at ClinicalTrials.gov (NCT03245216).

## Introduction

Parkinson’s disease (PD) is a neurodegenerative disorder characterized by a loss of dopaminergic neurons in the basal ganglia. Major motor symptoms include bradykinesia, tremor, rigidity, and postural instability, resulting in slowed and unsecure movement control (Jankovic, 2008). Consequently, these patients suffer from various limitations in daily life activities, reduced mobility and have a substantially increased risk of falling (Fasano et al., 2017). Besides their motor impairment, cognitive deficits are highly prevalent in PD patients, including disturbed visuospatial perception, executive, and memory functions (Moustafa et al., 2016). These cognitive deficits are strongly interrelated with motor symptoms (Lord et al., 2014; Kelly et al., 2015; Moustafa et al., 2016), and have also been associated with impaired motor learning in PD (Price and Shin, 2009; Vandenbossche et al., 2009).

Pharmacological treatment can improve patients’ motor impairments, however, response to dopaminergic therapy diminishes with time, and several features of motor control are refractory to pharmacological treatment (Galna et al., 2015). This highlights the importance of complementary non-pharmacological treatments with exercise and physiotherapy playing a key role in this context (Tomlinson et al., 2013; van der Kolk and King, 2013; Abbruzzese et al., 2016). As motor rehabilitation involves repeated practice of movement skills, it constantly induces motor learning processes (Abbruzzese et al., 2016). With respect to the ability to (re-)learn motor skills, PD patients demonstrate clear deficits compared to healthy adults of similar age (Nieuwboer et al., 2009; Clark et al., 2014). The pathophysiological basis of these deficits is attributable to the loss of dopamine in the caudal basal ganglia (Petzinger et al., 2013), which are heavily involved in learning motor skills (Hikosaka et al., 2002; Doyon et al., 2009). Corticostriatal circuits are not only crucial in the early phases of acquiring new skills, but also have high relevance for the early consolidation of motor memory (associative striatum), as well as the development of automaticity (sensorimotor striatum) (Doyon et al., 2009; Ashby et al., 2010). These later phases of motor skill retention are particularly impaired in PD, further emphasizing the role of the basal ganglia in motor memory consolidation (Doyon, 2008; Marinelli et al., 2017). Critically, motor learning is not improved by anti-Parkinson medication, which emphasizes the need for alternative treatment options (Marinelli et al., 2017).

These circumstances highlight the importance of developing methods to facilitate motor memory formation and consolidation in PD. Rapidly growing evidence suggests, that acute aerobic exercise (AEX) enhances motor skill learning in healthy adult populations (Taubert et al., 2015; Roig et al., 2016), when performed in close temporal proximity to motor practice (Statton et al., 2015; Thomas et al., 2016a). More specifically, short bouts of cardiovascular exercise, performed immediately prior to (Roig et al., 2012; Mang et al., 2014; Skriver et al., 2014; Statton et al., 2015; Snow et al., 2016; Stavrinos and Coxon, 2017) or following (Roig et al., 2012; Thomas et al., 2016a,b; Lundbye-Jensen et al., 2017; Dal Maso et al., 2018) motor skill practice have shown to improve motor skill acquisition (online learning) and consolidation (offline learning). Several underlying mechanisms on the molecular and systems level have been discussed (Taubert et al., 2015). These include increased arousal and cerebral blood flow following acute AEX, stronger expression of neurotrophic factors (e.g., brain-derived-neurotrophic-factor, BDNF), decreased intracortical inhibition, and enhanced corticospinal excitability (Taubert et al., 2015). Recently, first studies investigated the effects of an acute exercise bout in rehabilitation settings. Nepveu et al. (2017) found enhanced offline gains of a visuomotor tracking task in elderly stroke survivors when skill practice was followed by exercise on a recumbent stepper. In contrast, Charalambous et al. (2018) were not able to find beneficial effects on a motor adaptation task in stroke patients with a short 5-min bout of either treadmill walking or a total body exercise.

First pilot work in human PD patients showed improved motor memory function following 12 weeks of aerobic training (Duchesne et al., 2015, 2016), however, to date evidence on the facilitation effects of acute exercise on motor skill learning is lacking. Petzinger et al. (2013) proposed a model for motor skill practice and AEX working synergistically to induce neuroplasticity in PD. They suggest that exercise-induced mechanisms in the brain provide an optimal milieu for practicing and optimizing motor skills. Growing evidence from animal PD models support this link between exercise and increased neuroplasticity (Jakowec et al., 2016).

Consequently, we aimed at exploring the effects of a single bout of AEX on motor skill learning in early PD patients. For this, patients either performed a single bout of moderate-intensity cycling or seated rest immediately prior to practicing a novel balance task. We hypothesized that the acute exercise bout would lead to (i) larger performance improvement during skill acquisition (online learning), and (ii) increased motor skill consolidation (larger offline gains) after 24 h.

## Materials and Methods

This study was preregistered (Registration Number: NCT03245216) at ClinicalTrials.gov. The registration protocol is accessible at https://clinicaltrials.gov/ct2/show/NCT03245216.

### Participants

A total of 17 early to mid-stage PD patients volunteered to participate in this study (Supplementary Figure 1). Patients gave written informed consent prior to participation, and the study was approved by the local ethics committee (reference number: 125_17B) of the FAU Erlangen-Nürnberg, Germany. Patients gave written informed consent prior to study participation according to the declaration of Helsinki. Patients were eligible for participation if they had (i) a Hoehn and Yahr score of <3; (ii) a score of ≤1 in the UPDRS item ‘postural instability’; (iii) were able to stand and walk independently; and (iv) were not familiar with the balance platform. Criteria for exclusion were (i) higher level of cognitive impairment indicated by a score of <21 in the Montreal Cognitive Assessment (MoCA) (Dalrymple-Alford et al., 2010); (ii) other clinically relevant neurological, internal or orthopedic conditions besides Parkinsonism that would interfere with the exercise paradigm or motor learning task; (iii) musculoskeletal conditions or surgery 1 year before the study enrolment; (iv) smoking >10 cigarettes/day or drinking >6 cups of coffee/day or >50 g of alcohol (equivalent of two glasses of wine) consumption/day (Winter et al., 2007).

### Experimental Design

**FIGURE 1:**
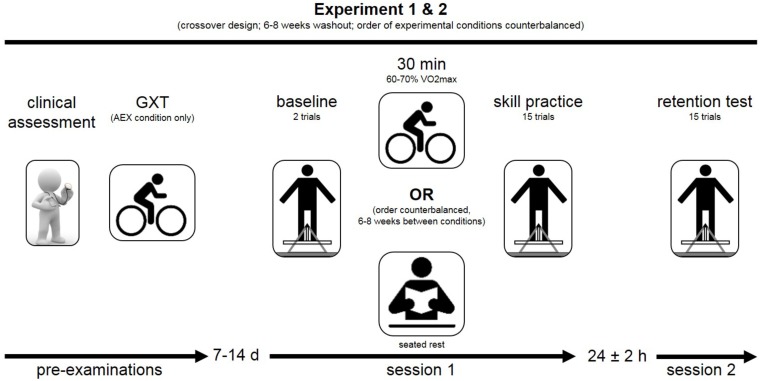
Schematic overview of the experimental design. Figure illustrates the experimental flow. Pre-examinations included a clinical assessment of the patients’ neurological and cognitive status. Subsequently, patients participated in two separate experiments, including either the experimental (cycling) or control condition (rest). The order of experiments was counter-balanced and separated by 6–8 weeks (cross-over design). Each of the two experiments included an acquisition phase (session 1), followed by a retention test (session 2) 24 h later. Cardiac screening included a graded exercise test (GXT) and was performed prior to the experiment including the cycling condition (AEX).

We implemented a crossover design in this study (Figure 1). Accordingly, patients participated in two separate experiments, requiring them to practice a motor skill preceded by one of two different experimental conditions: AEX or seated rest (REST). The order of experiments was counterbalanced, separated by a six to 8 weeks washout period. Patients were blinded to the researchers’ hypotheses regarding the effects of the different experimental conditions. We used block randomization stratified by gender (male/female) and age (<62/>62) to define the order of experimental conditions, since these two factors may modulate the effects of AEX on cognitive performance (Smith et al., 2005) and memory formation (Kamijo et al., 2009; Kramer and Colcombe, 2018). Each of the two experiments included (i) an acquisition session where the motor skill was practiced, followed by (ii) a retention test 24 ± 2 h later. PD patients were instructed to refrain from vigorous physical activity 48 h prior to and 24 h after the acquisition session, as well as from caffeine, alcohol and nicotine uptake 24 h before and after the acquisition session. Since the whole experimental routine was supervised by the same researcher (PW), blinding to the experimental condition was not possible.

Pre-examinations prior to each experiment included assessment of anthropometric and demographic data, motor function using the motor part of the Unified Parkinson’s Disease Rating Scale (MDS-UPDRS; Goetz et al., 2008), cognitive function using the MoCA (Dalrymple-Alford et al., 2010), and self-reported physical activity level using the IPAQ (Craig et al., 2003). All assessments were performed by a trained exercise therapist (PW) and with patients on medication.

After each motor practicing session (acquisition, retention), participants completed the National Aeronautics and Space Administration-Task Load Index (NASA-TLX) (Hart and Staveland, 1988). The NASA-TLX is a visual analog scale to assess the subjectively perceived workload of a task. It consists of six dimensions: mental demand, physical demand, temporal demand, performance, effort, and frustration. Each dimension can be rated on a twenty-step bipolar scale ranging from 0 (not demanding) to 20 (extreme demanding). Further, the average of the six dimensions can be calculated to determine the Raw Task Load Index (RTLX), which is a measure of the overall subjective workload (Hart, 2006). The NASA-TLX has been already used during learning of a balancing task and has shown to be useful to assess task difficulty (Akizuki and Ohashi, 2015).

Additional measures were taken in order to control for variables that might influence the motor learning process. This included a simple reaction time task (Deary et al., 2011) to test patients alertness after the experimental stimulus. Before and immediately following each experimental stimulus (AEX/REST), participants had to press a button as fast as possible with their index finger of the preferred hand when a cross appeared in a white box on the computer screen. A series of 20 presentations was given and reaction times averaged across trials. Additionally, sleep quality and daytime sleepiness were assessed using the Epworth Sleepiness Scale (Johns, 1991) and the Pittsburgh Sleep Diary (Monk et al., 1994).

### Motor Learning Task

We used a dynamic balancing task to examine patients’ motor learning performance. The stabilometer is widely used to study motor learning (Wulf et al., 2003; Lewthwaite and Wulf, 2010; Kiss et al., 2018), including PD populations (Chiviacowsky et al., 2012; Sehm et al., 2014). The device consists of a 107 × 65 cm wooden platform (stability platform; Lafayette Instrument Europe; Loughborough, United Kingdom), which is mounted on a fulcrum and has a maximum deviation of 15° to either side. Participants are required to stand with both feet on the platform and try to keep the platform as close to the horizontal as possible during each 30 s trial (Chiviacowsky et al., 2012). A millisecond timer measured time in balance, which is the time patients were able to keep the platform within ±5° from horizontal during each 30-s trial. Since the way this motor task is instructed may affect learning outcome (Wulf and Lewthwaite, 2009), standardized formulations were used to present the task to the participants, excluding any forms of motivation or the direction of attentional focus. We further provided rigidly standardized feedback on the achieved time in balance (knowledge of results) immediately after each trial but did not give any additional information on the patients’ performance strategy. Patients were secured with a safety harness and instructed to stand in a comfortable position (foot position was marked to ensure standardized positioning during all tests).

On the first day of each experiment (Figure 1), patients had a baseline block (2 trials) prior to the experimental condition (AEX, REST) in order to familiarize the patients to the task and to establish baseline performance. The acquisition phase started within 5 min after the experimental condition (immediately after the reaction time test). The acquisition phase included 15 practice trials (30 s), clustered into 5 blocks of 3 trials, with 60 s rest between trials and 120 s rest between blocks. The 24-h retention test included another 5 blocks of 3 trials (trial 1 of first block removed from data analysis to account for potential warm-up effects). All experimental session were supervised by the same researcher (PW), with patients on medication and at the same time of the day. The ‘on’ state was chosen in order to ensure that patients were able to securely perform the motor task, and in order to reduce the potential influence of motor symptoms (i.a., tremor).

### Exercise Protocol

Participants underwent cardiologic screening 7–14 days prior to the experiment. The procedure included a graded exercise test (GXT) performed on a stationary cycle ergometer and supervised by a cardiologist. Staring at 25 Watts and a constant pedal rate of ∼60 rpm, load was stepwise increased by 25 Watts every 3 min until exhaustion, and heart rate (HR) response and spirometry data (VO_2_/VCO_2_) were recorded continuously.

During the experiment, the AEX condition comprised a 5-min warm-up (intensity was progressively increased until target HR), followed by 25 min of pedaling at 60–80 rpm and a HR between 60 and 70% of pre-test HR at VO_2max_. Similar protocols have been used in studies of young healthy adults, and demonstrated improved motor skill learning (Chartrand et al., 2015; Statton et al., 2015; Snow et al., 2016).

Heart rate was continuously recorded using a HR monitor (Polar RS800). Additionally, patients’ subjective level of perceived exertion (Borg scale 6–20) and blood pressure was recorded every 3 min.

### Statistical Analysis

All statistical analyses were performed using IBM SPSS Statistics version 25.0 (IBM Corp; Armonk, NY, United States) and the alpha level set at *p* ≤ 0.05. Normality, variance homogeneity, and sphericity of the data were checked where appropriate.

#### Baseline Performance and Order Effects

We compared baseline performance between the two experimental conditions (AEX, REST) and between the two experiments (first, second) using independent samples *t*-tests. Similarly, we tested the immediate effect of AEX on skill performance by comparing changes from baseline to the first acquisition block between conditions (AEX, REST). The effect of AEX on alertness was explored by comparing reaction times changes (from pre- to post-exercise) between conditions using independent samples *t*-test.

#### Online and Offline Motor Skill Learning

The effect of AEX on motor skill learning was examined by separate mixed ANOVAs testing for the between-subject factor *Condition* (AEX, REST) and the within-subject factor *Blocks*. Since baseline skill levels (baseline block) were (i) higher at the second compared to the first experiment in this cross-over design (*T*_32_ = −3.003, *p* = 0.005), and (ii) significantly associated with online (*F*_1,31_ = 17.832; *p* < 0.001) skill gains, baseline performance was entered as a covariate into the model. Consequently, the effect of AEX on motor skill acquisition (online learning) was tested in a 2 (AEX, REST) × 5 (acquisition blocks 1–5) model. Effects of AEX on offline learning was analyzed in a 2 (AEX, REST) × 2 (acquisition block 5, retention block 1) model. Additionally, retention performance was tested in a 2 (AEX, REST) × 5 (retention blocks 1–5) model, including the last acquisition block (block 5) as covariate.

## Results

Baseline characteristics and exercise data of the 17 enrolled PD patients (13 males, 4 females) are presented in Table 1. The average workload during the AEX bout was 61.7 Watts (range: 11.0–93.1), with patients performing at a HR of 105 bpm (range: 76–129) and a RPE of 13.5 (range 11–15).

**Table 1:** Patient’s characteristics and exercise parameters.

			Experiment 1			Experiment 2			Graded exercise test			Aerobic exercise (AEX) Bout		
	Age range, yrs	IPAQ, MET-min/week	H and Y stage	UPDRS-III	MoCA	H and Y stage	UPDRS-III	MoCA	HF_max	Watt_max	VO_2max_, ml/min/kg	HR, bpm	Power output, Watt	RPE
**Patient 1**	60–65	8064.0	2.5	59.0	26.0	2.5	57.0	22.0	73.0	100.0	20.0	76.0	65.4	13.9
**Patient 2**	70–75	1596.0	2.0	22.0	24.0	2.5	20.0	25.0	146.0	100.0	27.1	118.0	86.6	14.1
**Patient 3**	55–60	1788.0	2.0	33.0	29.0	2.0	37.0	26.0	133.0	150.0	23.7	102.0	93.1	12.1
**Patient 4**	55–60	5295.0	2.0	14.0	28.0	2.0	17.0	24.0	151.0	175.0	29.0	129.0	89.2	13.2
**Patient 5**	75–80	1914.0	2.5	41.0	24.0	2.5	45.0	28.0	96.0	75.0	18.2	78.0	66.2	13.6
**Patient 6**	70–75	1515.3	2.5	27.0	28.0	2.5	27.0	28.0	115.0	100.0	28.8	96.0	75.0	13.8
**Patient 7**	60–65	1431.0	2.0	20.0	28.0	2.0	20.0	28.0	113.0	150.0	21.8	87.0	40.1	14.8
**Patient 8**	60–65	1908.0	2.0	29.0	26.0	2.0	27.0	26.0	114.0	50.0	14.3	91.0	12.8	13.9
**Patient 9**	65–70	2074.0	2.0	15.0	29.0	1.0	11.0	23.0	161.0	175.0	38.7	128.0	63.0	13.6
**Patient 10**	65–70	2059.5	2.0	17.0	27.0	2.0	17.0	27.0	130.0	125.0	20.4	98.0	54.6	14.6
**Patient 11**	60–65	2198.0	2.0	12.0	28.0	2.0	12.0	28.0	150.0	175.0	28.5	125.0	80.0	13.9
**Patient 12**	60–65	2133.0	2.0	11.0	28.0	2.0	11.0	28.0	126.0	125.0	17.1	117.0	10.8	13.3
**Patient 13**	60–65	2292.0	2.0	20.0	28.0	2.0	20.0	28.0	127.0	150.0	24.4	107.0	41.5	11.6
**Patient 14**	50–55	2826.0	2.0	15.0	23.0	2.0	15.0	23.0	165.0	225.0	35.4	125.0	55.6	13.6
**Patient 15**	65–70	15924.0	2.0	24.0	26.0	2.0	24.0	26.0	128.0	125.0	30.5	103.0	74.2	11.0
**Patient 16**	65–70	1554.0	2.0	15.0	28.0	2.0	15.0	28.0	120.0	75.0	20.1	96.0	80.0	13.9
**Patient 17**	55–60	1786.5	2.0	20.0	28.0	2.0	25.0	27.0	129.0	150.0	24.5	117.0	60.0	14.9
Mean	64.4	3315.2	2.1	23.2	26.9	2.1	23.5	26.2	128.1	130.9	24.9	105.5	61.7	13.5
SD	6.2	3656.9	0.2	12.2	1.8	0.3	12.5	2.1	23.1	44.7	6.5	17.1	24.2	1.1

There were no significant differences between the two experimental conditions in the NASA-TLX sum score (acquisition: *T*_32_ = −0.258, *p* = 0.798; retention: *T*_32_ = −0.947; *p* = 0.351) or any of its sub-categories (Table 2), suggesting that self-perceived workload during the balance task was not affected by AEX. Further, reaction time did not significantly change from pre- to immediately post exercise (*T*_16_ = 1.396, *p* = 0.182), and change scores were not different from the REST condition (*T*_32_ < 0.641, *p* = 0.526), indicating that alertness at the beginning of motor practice was not affected by AEX.

**Table 2:** Reaction time and perceived task demands.

	Aerobic exercise		Rest		
	Mean	*SD*	Mean	*SD*	*p*-value

**NASA-TLX**					
**Acquisition**					
Mental demand	9.4	5.9	8.5	5.4	0.53
Physical demand	10.6	4.5	10.5	4.3	0.96
Temporal demand	2.8	3.4	5.1	3.9	0.06
Performance	7.3	3.8	7.2	4.2	0.64
Effort	9.0	5.9	8.9	6.0	0.93
Frustration	3.5	3.6	4.3	4.8	0.83
*RTLX*	7.1	2.9	7.4	3.6	0.74
**Retention**					
Mental demand	7.9	5.3	7.7	5.1	0.92
Physical demand	8.3	4.5	10.4	3.5	0.28
Temporal demand	3.4	2.7	4.9	3.1	0.17
Performance	7.5	5.3	7.5	5.4	1.00
Effort	10.2	5.3	10.6	4.6	0.72
Frustration	3.4	3.3	5.9	5.0	0.20
*RTLX*	6.8	3.3	7.8	3.2	0.31

**Simple reaction time**					

Pre, ms	334.5	31.2	330.0	37.23	0.71
Post, ms	325.6	37.8	328.0	34.43	0.85
*Change from Pre, ms*	−8.9	26.1	−2.0	35.56	0.53

*Pre = prior to experimental stimulus (aerobic exercise, rest); Post = immediately following experimental stimulus (aerobic exercise, rest); RTLX = Raw Task Load Index.*

Balance performance at baseline (baseline block) did not differ significantly between the two experimental conditions (*T*_32_ = −0.753, *p* = 0.457). Further, changes in skill performance from the baseline block to the first practice block (*T*_32_ = 1.252, *p* = 0.220) were not affected by the acute AEX bout.

### Effects of Aerobic Exercise on Online Learning

**FIGURE 2:**
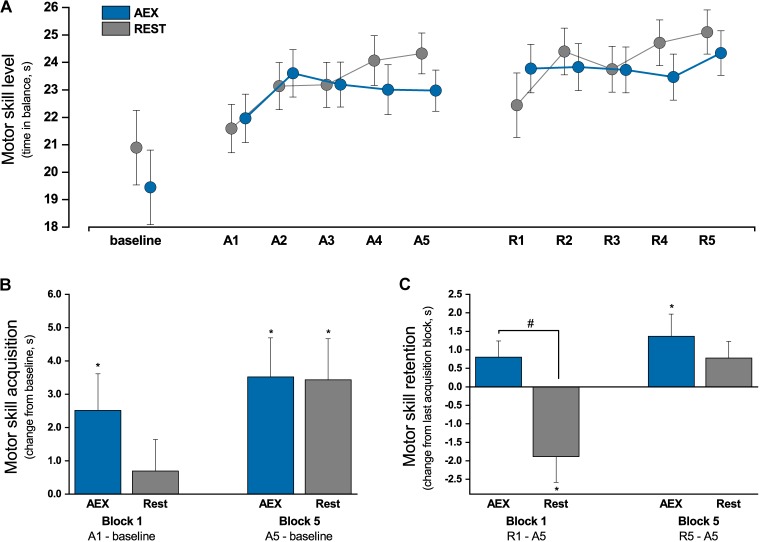
Motor skill performance. **(A)** Motor skill performance (time in balance) during acquisition (A1–A5) and retention (R1–R5); **(B)** online skill gains illustrated as change from baseline block to first (A1) and last (A5) acquisition block, ^∗^significantly different from baseline (paired *t*-test *p* ≤ 0.036); **(C)** motor skill retention illustrated as change from last acquisition block (A5) to first (R1) and last (R5) retention block, ^∗^significantly different from baseline (paired *t*-test *p* ≤ 0.036), ^#^significant difference between aerobic exercise (AEX) and REST condition (*F*_1,32_ = 10.734, *p* = 0.003); error bars indicate 1 SE.

A significant main effect for *Block* existed (*F*_4,124_ = 3.53, *p* = 0.009, η^2^ = 0.102), confirming that patients increased their balance time throughout the acquisition session (Figure 2A). The overall performance level during practice was similar in both experimental conditions, as indicated by the absence of a *Condition* effect (*F*_1,31_ < 1, *p* = 0.842). The skill improvement from baseline to the last acquisition block was comparable between the two conditions (AEX = 18.1%, REST = 16.4%; *T*_32_ < 1, *p* = 0.959), suggesting that AEX had no effect on absolute online learning gain (Figure 2B). A significant *Condition* × *Block* interaction (*F*_4,124_ = 2.73, *p* = 0.032, η^2^ = 0.081) indicated that the time course of skill improvements differed between the two experimental conditions. Contrasts suggest that AEX led to rapid initial performance increases, which were attenuated during late practice (block 4 vs. previous: *F*_1,31_ = 4.770, *p* = 0.037; block 5 vs. previous: *F*_1,31_ = 7.020, *p* = 0.013).

### Effects of Aerobic Exercise on Offline Learning

Offline learning was assessed by comparing change in balance performance from the last acquisition block to the first retention block (Figure 2C). There was no significant main effect for *Condition* (*F*_1,32_ < 1, *p* = 0.990) or *Block* (*F*_1,32_ = 1.736, *p* = 0.197). The significant *Condition* × *Block* interaction (*F*_1,32_ = 10.734, *p* = 0.003, η^2^ = 0.251) suggests that offline gains differed between conditions. *Posthoc* tests revealed that balance performance significantly decreased in the REST condition (−7.8%, *T*_16_ = −2.710, *p* = 0.015), while patients in the AEX condition sustained their performance level from practice to retention (+3.5%, *T*_16_ = 1.845, *p* = 0.84).

In addition to offline gains, we analyzed further skill changes during the retention session. A significant main effect for *Block* (*F*_4,124_ = 2.694, *p* = 0.045; η^2^ = 0.080) and a significant *Condition* × *Block* interaction (*F*_4,124_ = 3.048, *p* = 0.028; η^2^ = 0.090) existed. This suggests that patients continued to improve their balance time during the retention test, but differences existed between conditions. Contrasts revealed that skill gains during retention session were weaker in the AEX compared to the REST condition (block 1 vs. block 5: *F*_1,31_ = 5.312, *p* = 0.028). However, skill level (main effect *Condition*: *F*_1,31_ = 2.505, *p* = 0.124) and overall skill improvement from baseline were comparable between the AEX and REST condition (AEX = 25.1%, REST = 20.1%; *T*_32_ < 1, *p* = 0.721).

## Discussion

To our knowledge, this is the first study to examine whether a single bout of moderate-intensity cycling enhances (i) motor skill acquisition (online learning) or (ii) motor skill consolidation (offline learning) in PD patients. We found that the exercise bout had no effect on performance level or overall skill improvement during acquisition, thus not confirming our initial hypothesis. However, cycling seemed to affect the time course of skill change, which was expressed by larger initial and weakened late performance gains during acquisition (Figure 2A). In line with our hypothesis, offline learning was improved in the AEX condition, which indicates improved motor memory consolidation in these patients. This finding is of great importance, given that these patients have particular deficits in motor skill learning compared to healthy elderly of similar age.

### Effect of Aerobic Exercise on Online Motor Learning

We found that AEX did not improve online learning of PD patients. In fact, ANOVA results suggest that timing of improvements throughout practice was affected by AEX. Online gains appeared to be larger during the initial practice phase, and less pronounced during late practice (block 4 and 5) in the AEX condition. Since overall skill improvement during practice session, i.e., change from pre-exercise baseline to last acquisition block, was comparable between the AEX (+3.5 ± 4.8 s) and REST condition (+3.4 ± 5.1 s), AEX does not seem to negatively affect online skill learning.

Previous studies on the effect of a single bout of AEX on skill acquisition involved young healthy individuals, and findings were inconsistent. While some authors demonstrated increased online learning gains (Chartrand et al., 2015; Statton et al., 2015) or overall skill level (Perini et al., 2016), others reported no effects on motor skill acquisition (Singh et al., 2016; Snow et al., 2016). One study by Singh et al. (2016) reported worse online learning in the AEX group compared to a resting control group in a bimanual targeting task, and this was only seen for movement trajectory but not for response time and accuracy. Our study involved PD patients, and it is suggested that age and memory level may moderate the effect of AEX on online and offline motor learning (Roig et al., 2016). Thus, the results are difficult to relate directly to previous research. However, our data suggest no benefit of acute exercise bouts on online learning. More work on elderly and physically impaired populations is necessary, since data in this field is scarce.

*Post hoc* analyses indicated that the aerobic cycling reduced performance improvements during late practice (acquisition block 4–5). Inspection of the performance curves (Figure 2) indicated that the lower learning rates in the later blocks were likely attributed to rapid initial improvements during practice, as performance gains from baseline to the first acquisition blocks (block 1–2) appeared to be particularly high in the AEX condition. Several reasons may explain this finding. First, patients already demonstrated a high performance level in the balance task at baseline (AEX: 19.4 ± 5.1 s, REST: 20.9 ± 6.1 s). Consequently, potential for further improvement was limited, and the rapid initial skill gains in the AEX condition may have impeded additional improvement in the later phase of practice. Establishing baseline performance on the acquisition day may have also contributed to this fact. Further, results from the NASA-TLX indicated that patients perceived task difficulty relatively low compared to other studies (Akizuki and Ohashi, 2015). It is possible that the rapid increase during early practice in the AEX condition, in combination with a generally low level of difficulty, led to reduce task challenge, and consequently to a ceiling effect that may have prevented further online learning gains. Another explanation might be the specific characteristics of the study sample, as participants were older adults aged between 50 and 80 years. Given their Parkinson’s disease, they further presented moderate levels of motor impairment and a reduced exercise capacity. Thus, the limited learning gains particularly during late practice may have also been attributed to physical or mental fatigue. As both cycling and balancing involve similar muscle groups of the lower extremity, muscular fatigue may have negatively influenced performance during later practice trials, particularly the performance decline in blocks 3–5 of acquisition. In addition, the 30-min cycling exercise prior to skill practice may also have increased mental workload with increasing practice time. This seems plausible, since several patients demonstrated first signs of cognitive decline, which has been associated with increased mental effort in performing tasks and difficulties in maintaining concentration (Friedman et al., 2016). However, we found that patients’ self-reported level of mental or physical workload (NASA-TLX) throughout the practice session was moderate and did not differ between both conditions, thus making this explanation unlikely.

Noteworthy, all studies that found beneficial effects of an acute AEX bout on online learning applied a motor skill that required the control of only a few effectors (muscle groups). In contrast, the balancing task implemented in our trial requires the control of multiple effectors and the integration and coordination of extensive perceptual information. Previous studies investigating the effects of an acute AEX bout on learning of motor skills requiring the control of multiple effectors were also not able to find advantageous effects (Singh et al., 2016; Helm et al., 2017; Charalambous et al., 2018). As discussed in a meta-analysis by McMorris et al. (2015), the prefrontal cortex is mainly involved in the control of multiple effectors as well as the integration and coordination of perceptual information. Further, it has been reported that performance increase in the stabilometer task correlates mainly with changes in the prefrontal cortex (Taubert et al., 2010). Besides the beneficial effects of an acute AEX bout on learning related structures, the AEX has shown to have negative effects on prefrontal cortex activity, due to an increase in catecholamines (Arnsten, 2009, 2011). Consequently, the beneficial effects of an acute AEX bout on online learning may not apply for skills requiring a higher prefrontal cortex involvement.

### Effect of Aerobic Exercise on Offline Motor Learning

A single bout of moderate-intensity cycling substantially improved offline motor skill learning in PD, and the effect size indicated that this effect was large. While AEX resulted in preserved skill levels after 24-h, performance decreased significantly in the REST condition. With this finding, our data are in line with a great deal of evidence showing exercise-induced improvements in motor memory consolidation in healthy young individuals (Roig et al., 2012; Thomas et al., 2016a,b; Lundbye-Jensen et al., 2017; Dal Maso et al., 2018; Ferrer-Uris et al., 2018). Recently, similar findings have been reported in a sample of elderly stroke survivors (Nepveu et al., 2017). Consistently, these studies indicate that a single bout of AEX can positively affect motor memory consolidation following initial practice.

This finding has strong implications for clinical practice and neurorehabilitation, as the practice and (re-)learning of motor skills (e.g., gait, balance) is an essential component during all stages of motor rehabilitation (Abbruzzese et al., 2016). To the best of our knowledge, we were the first to demonstrate improved motor memory consolidation induced by a single bout of exercise in PD patients. Recently, Duchesne et al. (2015, 2016) showed improved motor memory function in PD patients following 12 weeks of aerobic training. However, the immediate effects of acute exercise bouts on skill learning have not yet been investigated in this population. The present data suggest that AEX is effective in enhancing motor memory consolidation in PD. PD patients have particular deficits in consolidation and switching to the autonomous stage of learning compared to healthy adults of similar age (Nieuwboer et al., 2009; Clark et al., 2014). This is attributed to the loss of dopamine in the caudal basal ganglia (Petzinger et al., 2013), which affects striatal structures involved in the early consolidation (associative striatum) and the development of automaticity (sensorimotor striatum) (Doyon et al., 2009; Beeler et al., 2013). Consequently, our data suggest that acute AEX is an effective strategy to counteract these deficits, which is very promising given the potential impact on motor rehabilitation.

Several mechanisms may explain the enhancing effects of acute exercise bouts on motor skill consolidation (i.e., offline learning). On the molecular level, increased catecholamine levels (i.e., dopamine, adrenalin, noradrenalin) have been demonstrated following acute bouts of AEX (Winter et al., 2007; Taubert et al., 2015), and were associated with improved learning outcomes in humans (Cahill and Alkire, 2003). Even more importantly from a PD perspective, dopamine is a key facilitator of neuroplasticity and memory (Hosp et al., 2011; Beeler et al., 2013; Rioult-Pedotti et al., 2015), and was recently suggested to be involved in the exercise-mediated increase of memory consolidation in healthy individuals (Winter et al., 2007; Mang et al., 2017). PD patients demonstrate disease-related dopamine depletion and associated dopamine-related aberrant motor learning (Beeler, 2011; Beeler et al., 2013). Thus, our findings may indicate an exercise-induced upregulation of patients’ dopamine levels, which would explain the improved learning outcome (Petzinger et al., 2013; Jakowec et al., 2016). This is supported by the work of Beeler (Beeler, 2011; Beeler et al., 2013), who suggested that skill practice during peak dopamine levels, as it would theoretically be the case immediately following acute AEX, might allow a relatively normal corticostriatal plasticity and consequently a relatively healthy motor learning behavior. However, since we were not able to assess dopamine levels or to test patients concurrently in ‘off’ medication state, this remains speculative and needs further exploration in future trials.

Additionally, AEX leads to an elevation in neurotrophic factors, such as BDNF, which are involved in the memory formation process (Knaepen et al., 2010; Szuhany et al., 2015; da Silva et al., 2016; Hirsch et al., 2018). Consequently, it has been suggested that the positive effect of AEX on memory consolidation may be linked to increased BDNF expression following exercise, thus promoting synaptic plasticity and long-term potentiation (Taubert et al., 2015). Further, BDNF is also heavily discussed in the context of neurorehabilitation in PD, since BDNF level increases and concurrent motor improvements have been found both, in PD animal models as well as humans in response to exercise (Svensson et al., 2015; Hirsch et al., 2018). Consequently, the enhanced skill consolidation found following AEX in the present study could have been the result of increased BDNF availability during the post-exercise phase. However, the relation between elevated neurotrophins after AEX and improved motor learning is ambiguous. While some studies were able to find a relationship in young adults (Skriver et al., 2014), others failed to find associations, even though reporting elevated BDNF levels after exercise bout (Mang et al., 2014; Helm et al., 2017; Charalambous et al., 2018). As BDNF seems to be connected to lactate concentrations in the blood (Rojas Vega et al., 2006; Rojas Vega et al., 2012), a dose-response relationship between AEX intensity and BDNF release is suggested. The fact that we found positive effects already with moderate-intensity AEX may indicate that this was sufficient to induce a considerable release of BDNF in PD patients, which would be supported by other studies documenting BDNF-response in neurological conditions (Gold et al., 2003). In addition to its trigger function, it has been suggested that the accumulated lactate due to the AEX can be metabolized by the brain and may serve as an energy source (Dalsgaard et al., 2004; Kemppainen et al., 2005).

On the systems-level of brain organization (Voss et al., 2013), our findings of improved offline learning might indicate an increased corticomotor excitability during skill practice. Exercise-increased blood lactate has been associated with enhanced corticospinal excitability (Coco et al., 2010), and this was associated with offline motor learning gains (Singh et al., 2014; Ostadan et al., 2016). However, findings are inconsistent, as some studies were not able to find changes in corticomotor excitability with AEX in healthy young adults or stroke survivors (McDonnell et al., 2013; Nepveu et al., 2017; Stavrinos and Coxon, 2017). Increased intra-cortical excitability induced by reduced GABA_A_ synaptic inhibition was also associated with offline gains in motor learning (Nepveu et al., 2017; Stavrinos and Coxon, 2017), which could be another explanation for the improved learning outcome in our study. Lastly, recent studies reported indicators of an exercise-induced increase in functional connectivity of corticomotor networks (Rajab et al., 2014), which were associated with improved motor skill retention (Dal Maso et al., 2018). The aforementioned findings suggest promising exercise-induced brain-level mechanisms, which need further investigation to be associated with improved motor learning outcome on the behavioral level.

### Limitations

Some limitations of this study need to be addressed. First, the 6-week washout phase of this cross-over trial was relatively short, and significant carry-over effects existed. Consequently, participants had a higher initial performance level during the second experiment (*T*_32_ = −3.003, *p* = 0.005), and this was associated with reduced skill improvement during practice (*F*_1,31_ = 17.832; *p* < 0.001). However, since we counterbalanced the order of experimental conditions, this is unlikely to have affected our findings. Rather, it seems possible that patients’ potential for further skill improvement in the second experiment was limited, which could have masked more pronounced effects. This would also explain the ceiling effects that seemed to be present in this study. Patients’ physical activity status and aerobic capacity varied substantially (IPAQ range: 1,431.0–15,924.0 MET-min/week; VO_2max_ range: 17.1–35.4 ml/min/kg), which has been suggested to influence the effects of acute AEX on skill learning. These differences in fitness level could have led to variations in exercise response and subsequent molecular mechanisms (e.g., expression of catecholamines or neurotrophins). On a similar note, autonomic dysfunction is common in PD, and patients present reduced peak responses in graded exercise testing (Kanegusuku et al., 2016), which complicates accurate regulation of exercise intensity. This could have caused additional variance in individuals’ physiologic response to AEX and subsequent effects on motor skill learning. The assessment of additional physiological parameters (e.g., lactate, VO_2_) would have been desirable, but was not possible in this study. Lastly, since the learning task and the AEX bout (cycling) involve similar muscle groups, this may have caused fatigue effects in these muscles potentially explaining the performance decline during late acquisition (blocks 3–5). Future studies should implement upper extremity learning tasks to exclude this potential source of bias.

## Conclusion

Results from this pilot study suggest that a single bout of AEX can effectively enhance motor skill consolidation in PD patients. This was evidenced by improved offline gains 24 h after acquisition, when skill practice was preceded by 30 min of moderate-intensity cycling. Our results are promising, since they demonstrate that (re-)learning of motor skills in patients with existing motor control and learning deficits can be optimized by short bouts of exercise. The regular practice of motor skills is a prerequisite for maintaining independence and mobility in PD patients and a core principle in neurorehabilitation, and our data suggest a promising and convenient strategy to facilitate this process. More work is needed to understand the underlying mechanisms, to explore the effects of different exercise scheduling (i.a., timing, intensity), and to investigate the potential for PD patients at later disease stages.

## Author Contributions

SS and PW were responsible for conception and design of the study. JK and JW recruited patients and provided clinical advice. PW performed data acquisition and data processing. WA provided statistical support for data analysis. SS analyzed the data and wrote the first manuscript draft. PW, JK, JW, and KP critically discussed findings and reviewed the manuscript from a movement science, clinical, or statistical perspective. All authors gave final approval of the version to be submitted.

## Conflict of Interest Statement

SS, PW, and KP have received institutional research grants from Deutsche Stiftung Neurologie (German Foundation Neurology). KP has received institutional research grants from the Federal Ministry of Education and Research (BMBF), the German Statutory Pension Insurance, the Bavarian Virtual University German Foundation Neurology as well as compensation or honorary for serving on scientific advisory boards, lecturing or reviewing from AbbVie Gmbh, German Statutory Pension Insurance, BZgA, MS-Franken, Landkreis ERH, and ICF. JW reports personal fees outside of the submitted work from Teva GmbH, Ever Pharma GmbH, Desitin Arzneimittel GmbH, AbbVie GmbH & Co., KG, Biogen GmbH, and GlaxoSmithKline GmbH & Co., KG. JK holds ownerships of Portabiles HealthCare Technologies GmbH and Portabiles GmbH, received compensation and honoraria from serving on scientific advisory boards for Licher MT GmbH, AbbVie GmbH, UCB Pharma GmbH, GlaxoSmithKline GmbH & Co., KG, Athenion GmbH, and Thomashilfen GmbH; as well as lecturing from UCB Pharma GmbH, TEVA Pharma GmbH, Licher MT GmbH, Desitin GmbH, AbbVie GmbH, Solvay Pharmaceuticals, and Ever Neuro Pharma GmbH. The remaining author declares that the research was conducted in the absence of any commercial or financial relationships that could be construed as a potential conflict of interest.
