# Supplementary material for: A Single Bout of Aerobic Exercise Improves Motor Skill Consolidation in Parkinson’s Disease
Source: Front Aging Neurosci. 2018 Oct 22;10:328. doi: 10.3389/fnagi.2018.00328 (PMC6204491; doi:10.3389/fnagi.2018.00328)
Supplement: Supplementary file 1 [file Data_Sheet_1.doc]

**Supplementary Figure 1. Participants’ flow**

Assessed for eligibility (n= 28)

**Experiment I**

**Experiment II**

**Enrollment**

Excluded (n= 10)

♦ Not meeting inclusion criteria (n= 5)

♦ Declined participation (n= 5)

**Rest Condition (n= 9)**

♦ Lost to experiment II (n=0)

**Aerobic Exercise (n= 9)**

♦ Received allocated intervention (n= 9)

♦ Discontinued intervention (n= 0)

**Aerobic Exercise (n= 8)**

♦ Lost to experiment II (n= 0)

**Rest Condition (n= 9)**

♦ Received allocated intervention (n= 9)

♦ Discontinued intervention (n= 1)

- non-related adverse event (n= 1)

Randomized (n= 18)
